# Diagnostic Conversion From Psychotic Unipolar Depression to Bipolar and Psychotic Disorders: A Swedish Registry‐Based Study

**DOI:** 10.1111/acps.70059

**Published:** 2025-12-17

**Authors:** Ahmed Al‐Wandi, Mikael Landén, Axel Nordenskjöld

**Affiliations:** ^1^ Faculty of Medicine and Health University Health Care Research Centre, Örebro University Örebro Sweden; ^2^ Institute of Neuroscience and Physiology The Sahlgrenska Academy at Gothenburg University Gothenburg Sweden; ^3^ Department of Medical Epidemiology and Biostatistics Karolinska Institutet Stockholm Sweden

**Keywords:** diagnostic conversion, diagnostic stability, psychotic depression

## Abstract

**Objective:**

To estimate the cumulative incidence of diagnostic conversion from psychotic unipolar depression to bipolar and psychotic disorders in Sweden.

**Methods:**

Data from Swedish national registers were used to identify incident cases of psychotic unipolar depression between 2005 and 2011. To minimize the risk of misclassification, patients with a prior history of psychotic disorders, bipolar disorders, or manic episodes were excluded. Patients were followed until the first occurrence of a diagnostic change, death, or December 31, 2021. The cumulative incidence of diagnostic change was estimated using a competing risk model.

**Results:**

A total of 7836 patients diagnosed with psychotic depression between 2005 and 2011 were included. The median age at index diagnosis of psychotic depression was 49 years (interquartile range: 35–65), and 56.7% were women. By the end of follow‐up, 28.8% (95% CI: 27.7–29.9) of patients had undergone a diagnostic change to either a psychotic or bipolar disorder. The cumulative incidence of conversion was higher to psychotic disorders (17.5%, 95% CI: 16.6–18.4) than to bipolar disorders (14.7%, 95% CI: 13.8–15.5). In a sensitivity analysis requiring at least two recorded diagnoses separated by ≥ 1 year, the overall incidence of diagnostic change decreased to 19.0% (95% CI: 18.1–20.0), with corresponding rates of 10.0% (95% CI: 9.3–10.7) to psychotic disorders and 9.8% (95% CI: 9.1–10.5) to bipolar disorders. Diagnostic conversion was more common among younger individuals.

**Conclusion:**

Approximately 20%–30% of patients diagnosed with psychotic depression in secondary care in Sweden are expected to receive a subsequent diagnosis of a bipolar or psychotic disorder within 17 years. This has important clinical implications, as prognosis and treatment strategies differ between these conditions. Further research is needed to identify risk factors for diagnostic conversion to improve early detection and management.

## Key Points

- Significant Outcomes
  ○ Within 17 years of follow‐up, approximately 20%–30% of patients diagnosed with psychotic depression in Sweden are subsequently diagnosed with a bipolar and/or psychotic disorder.
  ○ Diagnostic conversion to psychotic disorders was somewhat more common than to bipolar disorders. However, this difference was minimal in a sensitivity analysis requiring at least two recorded diagnoses separated by ≥ 1 year, with both outcomes showing a cumulative incidence of approximately 10%.
  ○ Overall, diagnostic conversion was more common among younger individuals.
- Limitations
  ○ Limited diagnostic reliability of bipolar and psychotic disorders may result in both underdiagnosis and misdiagnosis, thereby introducing uncertainty in estimates of the true diagnostic conversion rates.
  ○ Incomplete coverage of primarily private outpatient and primary care data may have resulted in some diagnosed cases being missed.

## 1 Introduction

Psychotic unipolar depression refers to a depressive episode accompanied by psychotic symptoms, including hallucinations, delusions, or depressive stupor [1]. It may be a clinical manifestation of another disorder diagnosed at a later stage [1]. Psychotic depression has consistently been identified as a risk factor for subsequent development of bipolar disorders among patients with unipolar depression [2, 3, 4]. Several registry‐based studies have reported a conversion rate of 7%–8% to bipolar disorders in patients initially diagnosed with psychotic depression over follow‐up periods of up to 12–15 years [5, 6, 7].

The conversion rate from psychotic unipolar depression to other psychiatric disorders, such as schizophrenia or schizoaffective disorder, is not well studied, which is of importance since prognosis and treatment strategies differ across disorders. For example, antipsychotics are considered a first‐line treatment for schizophrenia but have questionable efficacy during the maintenance phase of psychotic unipolar depression [8, 9, 10]. Since antipsychotics are associated with various adverse effects, it is important to ensure that the patient gains a clear benefit from the medication [11].

Furthermore, schizophrenia is generally associated with a poorer prognosis than psychotic depression [12]. Early intervention in schizophrenia could potentially improve long‐term outcomes [13], which underscores the importance of recognizing that psychotic depression may, in some cases, represent the initial clinical manifestation of schizophrenia.

In summary, there is a notable lack of research on the conversion rate from psychotic depression to other psychiatric disorders. Further studies are needed to address this gap, given the important implications of accurately assessing prognosis and optimizing treatment strategies.

## 2 Aims of the Study

The aim of this study was to estimate the cumulative incidence of diagnostic conversion from psychotic unipolar depression to bipolar and psychotic disorders in Sweden using national registry data.

## 3 Methods

All patients with a primary diagnosis of psychotic unipolar depression, recorded in the Swedish National Inpatient or Outpatient Register between 2005 and 2011, were followed until the first occurrence of diagnostic change, death, or December 31, 2021. The cumulative incidence of diagnostic change was then calculated using a competing risk model.

### 3.1 Sources

The Swedish Inpatient Register has covered at least 98% of all psychiatric discharges since 1986. Specialized outpatient visits are registered in the Swedish Outpatient Register; however, data from primary care visits are not included. The Outpatient Register has a lower coverage of approximately 80%, primarily due to incomplete data from private healthcare providers [14]. Diagnostic codes from the tenth edition of the International Classification of Diseases (ICD‐10) were used to identify individuals with psychotic unipolar depression, excluding those with prior diagnoses of psychotic disorders, bipolar disorders, or manic episodes. Specific ICD‐10 codes used in the analysis are provided in Appendix A.

We extracted data on deaths from the Swedish Cause of Death Register, which has recorded almost every death in Sweden since 1952 [15].

Statistics Sweden linked data from the abovementioned registers using personal identity numbers, which are given to all permanent residents in Sweden [16].

### 3.2 Main Analysis

All patients with a first‐time main diagnosis of psychotic unipolar depression between 2005 and 2011 were identified from the Inpatient and Outpatient Registers. Patients were excluded if they had a main or ancillary diagnosis of a psychotic disorder, bipolar disorder, or manic episode either prior to or overlapping with the index episode, or if they had a prior diagnosis of psychotic unipolar depression from 1997 in the Inpatient Register and 2001 in the Outpatient Register.

The patients were followed until the first recorded change in main diagnosis, death, or December 31, 2021. The cumulative incidence of transitions in main diagnosis to psychotic disorders and bipolar disorders was estimated using a competing risk model, with a follow‐up time of up to 17 years. In this model, death was considered a competing event, as deceased individuals are no longer at risk of experiencing a diagnostic change. Separate models were used for each group of disorders. Therefore, a patient diagnosed with a psychotic disorder during follow‐up was not excluded from the analysis of bipolar disorders. Similarly, patients diagnosed within one subcategory (for instance, schizophrenia) were not excluded from the analyses of the other subcategories (for instance, acute and transient psychotic disorders). Thus, a patient could be included in multiple analyses. The cumulative incidence was calculated for the entire sample and stratified by age and sex.

SAS 8.3 was used for data extraction, and the cumulative incidence function (CIF) was estimated using R software [17, 18].

In addition to the CIF, we also calculated point estimates of the conversion rate using 1 minus the Kaplan–Meier estimate (1‐KM) and crude proportions.

### 3.3 Sensitivity Analyses

To minimize potential overestimation from low diagnostic reliability, we included only cases with the same diagnosis recorded on at least two occasions ≥ 1 year apart. For broader categories (e.g., psychotic or bipolar disorders), any diagnosis within the category was sufficient (e.g., first and last diagnoses could differ); for subcategories, both diagnoses were required to fall within the same subcategory. Because at least 1 year of potential follow‐up was necessary for a repeated diagnosis to be observed, follow‐up in this analysis was censored on December 31, 2020. All other methodological procedures were identical to those in the main analysis.

We also performed a more stringent sensitivity analysis, excluding patients who, after their first transitional diagnosis, were later rediagnosed with psychotic depression.

Finally, using a longer washout period for inpatient care could introduce differential misclassification, as more prevalent outpatient cases might be included relative to inpatient cases. Thus, in a sensitivity analysis, we applied the same washout period from 2001 to both the Inpatient and Outpatient Registers.

## 4 Results

A total of 7836 patients were identified with a primary diagnosis of psychotic unipolar depression between 2005 and 2011. The median age at index diagnosis was 49 years (interquartile range [IQR]: 35–65 years), with women comprising 56.7% of the sample. Nearly half (47.1%) received their initial diagnosis of psychotic depression in outpatient care. The patient characteristics at baseline are presented in Table 1.

**TABLE 1:** Baseline characteristics of patients.

	All		Male		Female	
	*n*	%	*n*	%	*n*	%
All	7836	100.0%	3390	43.3%	4446	56.7%
0–25 years	1034	13.2%	424	12.5%	610	13.7%
26–50 years	3029	38.7%	1335	39.4%	1694	38.1%
51–75 years	2812	35.9%	1319	38.9%	1493	33.6%
> 75 years	961	12.3%	312	9.2%	649	14.6%
Index diagnosis in outpatient setting	3692	47.1%	1563	46.1%	2129	47.9%

Over a median follow‐up time of 10.6 years (IQR: 3.0–13.3 years), 2186 patients (27.9%) experienced a diagnostic shift, 1743 (22.2%) died during follow‐up, and 3907 (49.9%) completed the follow‐up period without a diagnostic change or death. Among patients who experienced a diagnostic shift, 974 (44.6%) were exclusively treated in outpatient care during follow‐up.

**FIGURE 1:**
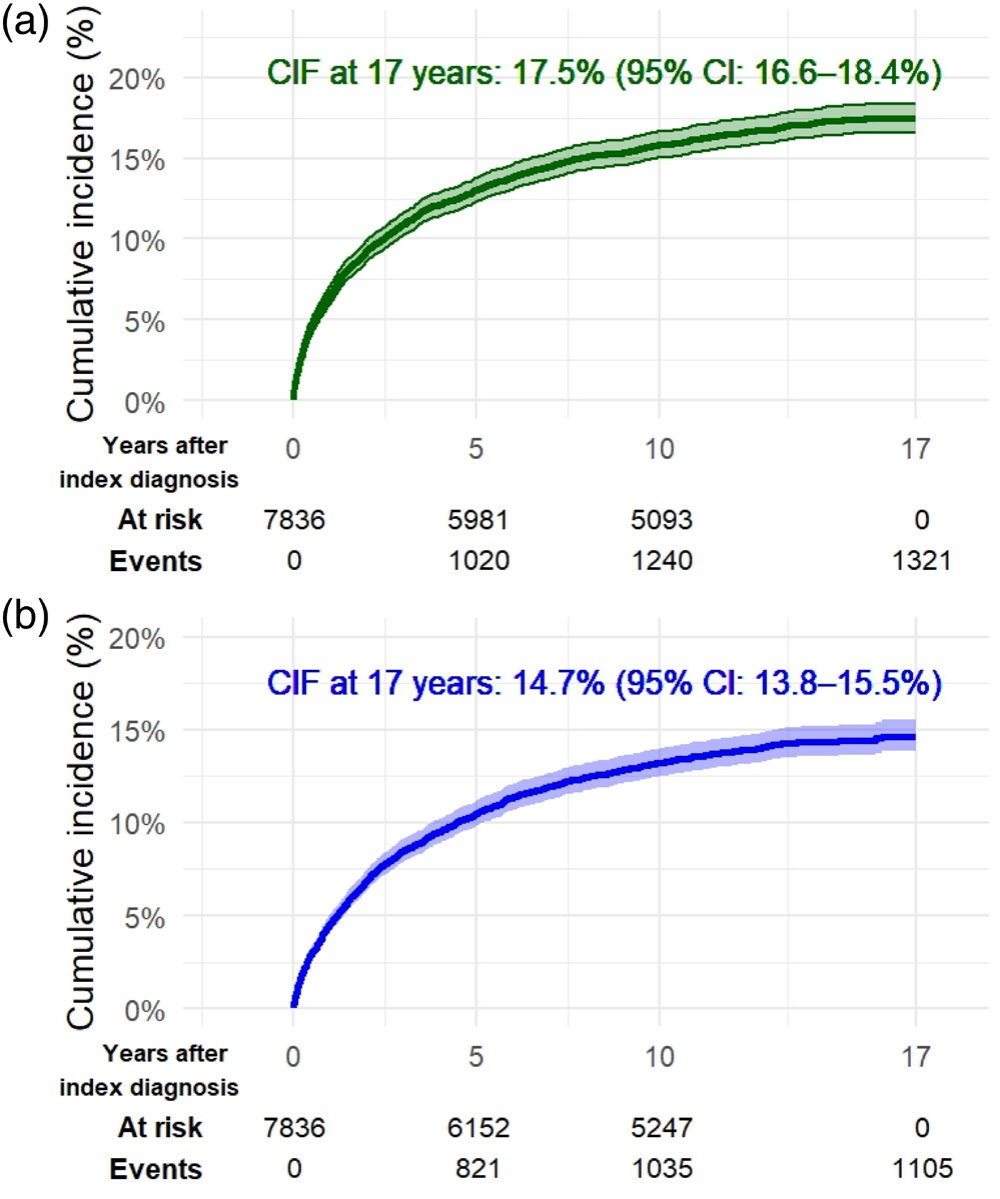
Cumulative incidence of diagnostic shift to (a) psychotic disorders and (b) bipolar disorders. CIF, cumulative incidence function.

**FIGURE 2:**
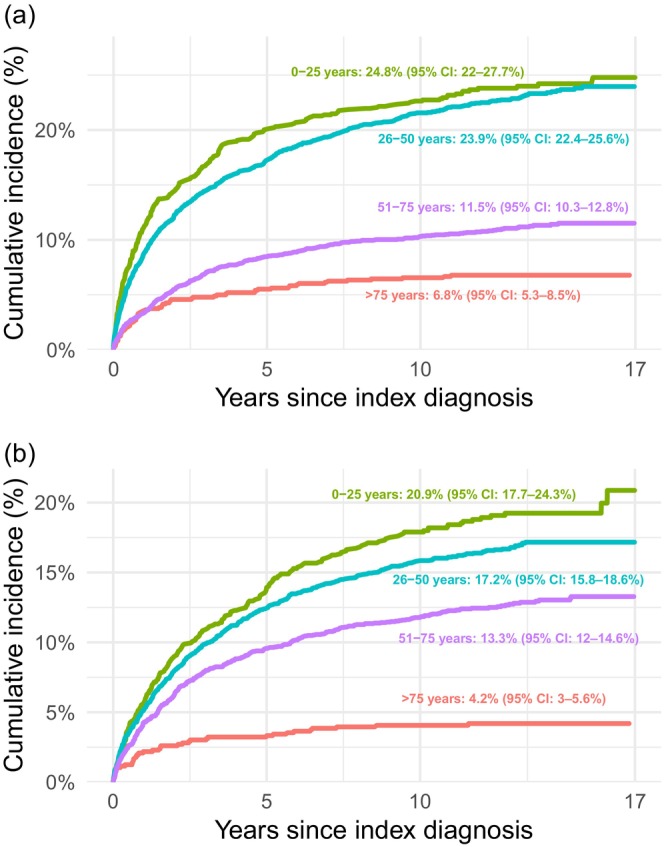
Cumulative incidence of diagnostic shift to (a) psychotic disorders and (b) bipolar disorders stratified by age.

The cumulative incidence of a diagnostic shift to psychotic and/or bipolar disorders after 17 years of follow‐up was 28.8% (95% confidence interval [CI]: 27.7–29.9) for the entire sample. Younger individuals had a higher cumulative incidence, which gradually decreased with age, ranging from 38.3% (95% CI: 34.6–42.0) in patients aged ≤ 25 years to 10.5% (95% CI: 8.7–12.6) in those > 75 years. The cumulative incidence of bipolar and psychotic disorders is presented for the entire study population in Figure 1a,b and stratified by age in Figure 2a,b. Table 2 presents the cumulative incidence of psychotic and bipolar disorders, combined and separately, overall and stratified by age and sex. Point estimates based on 1‐KM and crude proportions were generally consistent with the CIF, with 1‐KM typically yielding the highest values and proportions the lowest. Results for 1‐KM, CIF, and crude proportions from both the main and sensitivity analyses are presented in Appendix B.

**TABLE 2:** Cumulative incidence of diagnostic conversion to psychotic and bipolar disorders, combined and separately, stratified by age and sex.

Age	All	Male	Female
*Psychotic and bipolar disorders combined*			
All	28.8% (27.7–29.9)^a^	27.6% (25.9–29.2)	29.8% (28.4–31.2)
0–25 years	38.3% (34.6–42.0)	43.6% (34.8–52.0)	35.6% (31.8–39.4)
26–50 years	36.5% (34.7–38.3)	34.8% (32.2–37.4)	37.8% (35.4–40.3)
51–75 years	23.3% (21.7–24.9)	19.7% (17.5–21.9)	26.5% (24.1–28.9)
> 75 years	10.5% (8.7–12.6)	9.9% (6.9–13.6)	10.8% (8.6–13.4)
*Psychotic disorders*			
All	17.5% (16.6–18.4)	17.3% (16.0–18.6)	17.7% (16.5–18.9)
0–25 years	24.8% (22.0–27.7)	29.4% (25.0–34.0)	21.5% (18.0–25.3)
26–50 years	23.9% (22.4–25.6)	23.8% (21.5–26.2)	24.1% (21.9–26.3)
51–75 years	11.5% (10.3–12.8)	9.5% (7.9–11.2)	13.3% (11.6–15.2)
> 75 years	6.8% (5.3–8.5)	5.8% (3.5–8.7)	7.3% (5.4–9.5)
*Bipolar disorders*			
All	14.7% (13.8–15.5)	13.2% (11.8–14.5)	15.8% (14.7–17.0)
0–25 years	20.9% (17.7–24.3)	18.6% (12.4–25.9)	22.7% (19.4–26.1)
26–50 years	17.2% (15.8–18.6)	14.9% (13.0–16.9)	19.0% (17.1–20.9)
51–75 years	13.3% (12.0–14.6)	11.8% (10.1–13.7)	14.5% (12.7–16.5)
> 75 years	4.2% (3.0–5.6)	4.2% (2.3–6.8)	4.2% (2.8–6.0)

^a^ Values in parentheses indicate 95% confidence intervals.

### 4.1 Diagnostic Shift to Psychotic Disorders

The cumulative incidence of diagnostic shift to psychotic disorders was 17.5% (95% CI: 16.6–18.4), with minimal difference between males and females in the total sample. However, when stratified by age, the conversion rate was higher among males aged ≤ 25 years. In the other age groups, incidence was somewhat higher among females.

In total, 42.5% of patients diagnosed with psychotic disorders received treatment exclusively in outpatient care.

Among specific psychotic disorders, conversion to delusional disorder was the most common, with a cumulative incidence of 5.0% (95% CI: 4.5–5.5), followed by schizoaffective disorder at 3.7% (95% CI: 3.3–4.2), and schizophrenia at 3.3% (95% CI: 2.9–3.8). The category “other psychotic disorders” was more prevalent than any of the specific disorders, with a cumulative incidence of 8.9% (95% CI: 8.2–9.5). The majority of cases in this group (87.5%) were diagnosed with unspecified psychosis (F29).

Table 3 presents the cumulative incidence of psychotic disorder subcategories overall and stratified by age and sex.

**TABLE 3:** Cumulative incidence of diagnostic conversion to psychotic disorder subcategories and bipolar disorder type 1, stratified by age and sex.

Age	All	Male	Female	All	Male	Female
*Schizophrenia*				*Delusional disorder*		
All	3.3% (2.9–3.8)^a^	4.1% (3.4–4.8)	2.7% (2.3–3.3)	5.0% (4.5–5.5)	5.0% (4.3–5.8)	4.9% (4.2–5.7)
0–25 years	7.5% (5.8–9.4)	11.0% (8.2–14.4)	5.0% (3.2–7.4)	3.2% (2.3–4.4)	4.0% (2.4–6.2)	2.7% (1.6–4.2)
26–50 years	5.2% (4.5–6.1)	6.3% (5.1–7.8)	4.4% (3.4–5.5)	5.8% (5.0–6.7)	6.4% (5.1–7.8)	5.3% (4.3–6.5)
51–75 years	0.9% (0.6–1.3)	0.6% (0.3–1.1)	1.2% (0.8–1.9)	5.5% (4.5–6.5)	4.4% (3.4–5.7)	6.4% (5.0–8.0)
> 75 years	No cases	No cases	No cases	2.7% (1.8–3.9)	3.2% (1.6–5.6)	2.5% (1.5–3.9)
*Schizoaffective disorder*				*Bipolar disorder type 1*		
All	3.7% (3.3–4.2)	3.3% (2.7–4.0)	4.1% (3.5–4.8)	2.9% (2.5–3.4)	3.1% (2.5–3.9)	2.8% (2.3–3.3)
0–25 years	7.2% (5.7–8.9)	7.9% (5.4–11.0)	6.8% (5.0–9.0)	5.0% (3.4–7.1)	6.2% (2.8–11.4)	4.3% (2.9–6.2)
26–50 years	5.7% (4.9–6.7)	4.6% (3.5–5.9)	6.6% (5.4–8.1)	3.5% (2.9–4.2)	3.4% (2.5–4.5)	3.6% (2.8–4.6)
51–75 years	1.6% (1.1–2.1)	1.3% (0.7–2.2)	1.8% (1.2–2.6)	2.2% (1.7–2.8)	2.4% (1.6–3.3)	2.1% (1.5–3.0)
> 75 years	0.1% (0.0–0.6)	No cases	0.2% (0.0–0.8)	0.9% (0.5–1.7)	1.3% (0.4–3.1)	0.8% (0.3–1.7)
*Acute and transient psychotic disorders*				*Other psychotic disorders*		
All	5.1% (4.6–5.7)	4.3% (3.5–5.2)	5.7% (5.0–6.5)	8.9% (8.2–9.5)	9.1% (8.1–10.1)	8.7% (7.8–9.6)
0–25 years	10.2% (7.9–12.9)	10.8% (6.7–15.9)	9.8% (7.2–12.9)	14.8% (12.7–17.2)	17.3% (13.8–21.2)	13.1% (10.5–16.0)
26–50 years	7.1% (6.1–8.1)	5.8% (4.6–7.3)	8.0% (6.7–9.5)	12.3% (11.1–13.6)	13.3% (11.5–15.2)	11.6% (10.0–13.4)
51–75 years	2.3% (1.8–3.0)	1.4% (0.9–2.2)	3.1% (2.3–4.2)	4.9% (4.1–5.8)	3.9% (2.9–5.0)	5.9% (4.7–7.2)
> 75 years	1.7% (1.0–2.6)	1.3% (0.4–3.1)	1.8% (1.0–3.1)	3.0% (2.1–4.3)	1.9% (0.8–4.0)	3.6% (2.3–5.2)

^a^ Values in parentheses indicate 95% confidence intervals.

### 4.2 Diagnostic Shift to Bipolar Disorders

The cumulative incidence of diagnostic shift to bipolar disorders was 14.7% (95% CI: 13.8–15.5): 15.8% (95% CI: 14.7–17.0) in females and 13.2% (95% CI: 11.8–14.5) in males. When stratified by age, diagnostic conversion was higher among females across all age groups, except in patients > 75 years, where no difference was observed. Approximately half (49.3%) of patients diagnosed with bipolar disorders were treated exclusively in outpatient care. Less than one‐third (29.5%) had a registered diagnosis of either a hypomanic or manic episode during follow‐up.

The cumulative incidence of an established diagnosis of bipolar disorder type 1 (defined as any occurrence of a manic episode following an index episode of psychotic unipolar depression) was 2.9% (95% CI: 2.5–3.4). There were no major sex differences across age groups. The cumulative incidence of bipolar disorder type 1, overall and stratified by age and sex, is presented in Table 3.

### 4.3 Diagnostic Shifts Based on Initial Inpatient Versus Outpatient Depression Episode

The cumulative incidence of diagnostic shifts was slightly higher among patients whose index episode of psychotic depression was diagnosed in inpatient care. For psychotic and/or bipolar disorders combined, the rates were 29.9% (95% CI: 28.4–31.4) for inpatient care versus 27.5% (95% CI: 26.0–29.0) for outpatient care. For psychotic disorders, the rates were 17.7% (95% CI: 16.5–18.9) versus 17.2% (95% CI: 16.0–18.5), and for bipolar disorders, 16.0% (95% CI: 14.8–17.2) versus 13.1% (95% CI: 12.0–14.3).

### 4.4 Sensitivity Analyses

When the same diagnosis was required to be recorded at least twice, ≥ 1 year apart, the cumulative incidence decreased from 28.8% to 19.0% (95% CI: 18.1–20.0) for psychotic and/or bipolar disorders, from 14.7% to 9.8% (95% CI: 9.1–10.5) for bipolar disorders, and from 17.5% to 10.0% (95% CI: 9.3–10.7) for psychotic disorders. The decline in psychotic disorders may be partly expected, as acute and transient psychotic disorders are, by definition, not continuous. Accordingly, the conversion rate to acute and transient disorders dropped from 5.1% to 1.4% (95% CI: 1.1–1.7). Among the remaining groups of psychotic disorders, the largest decrease was observed in the category of other psychotic disorders, which declined from 8.9% to 3.6% (95% CI: 3.1–4.0), followed by delusional disorder, from 5.0% to 2.4% (95% CI: 2.1–2.8), schizophrenia, from 3.3% to 2.0% (95% CI: 1.7–2.4), and schizoaffective disorder, from 3.7% to 2.7% (95% CI: 2.3–3.1). The sensitivity analysis, overall and stratified by age and sex, is presented in Appendix C.

In the more stringent sensitivity analysis, excluding patients rediagnosed with psychotic depression, the cumulative incidence decreased compared with the sensitivity analysis described above, from 19.0% to 14.7% for psychotic and/or bipolar disorders, from 9.8% to 8.4% for bipolar disorders, and from 10.0% to 7.4% for psychotic disorders. The absolute difference for the subcategories of psychotic disorders was ≤ 0.5%, except for other psychotic disorders (0.9%).

Shortening the exclusion period for the Inpatient Register from 1997 to 2001, to match the Outpatient Register, had minimal impact on the results. The cumulative incidence increased only slightly: from 28.8% to 29.2% for psychotic and/or bipolar disorders, from 17.5% to 17.6% for psychotic disorders, and from 14.7% to 15.0% for bipolar disorders. No substantial differences were observed for the subcategories.

## 5 Discussion

We followed 7836 patients with an index episode of psychotic depression up to 17 years (median 10.6 years) in Swedish national registers. During the follow‐up period, the cumulative incidence of being diagnosed with either a bipolar or psychotic disorder was estimated at 28.8% (95% CI: 27.7–29.9). Specifically, 17.5% (95% CI: 16.6–18.4) converted to a psychotic disorder, and 14.7% (95% CI: 13.8–15.5) converted to a bipolar disorder. In a sensitivity analysis requiring at least two recorded diagnoses separated by ≥ 1 year, the conversion rates decreased to 19.0% for bipolar and/or psychotic disorders, 10.0% for psychotic disorders, and 9.8% for bipolar disorders.

Previous large‐scale studies have primarily focused on the transition to bipolar disorders, where registry‐based studies conducted in Denmark, Finland, and England have reported a conversion rate of approximately 7%–8% within a follow‐up period of up to 12–15 years [5, 6, 7]. A study from Korea by Kim et al. reported a crude conversion rate of 12.3% in a cohort of patients aged 19–34, with a follow‐up period of up to 6.5 years [19].

Our study demonstrating a higher conversion rate to bipolar disorders than these studies is somewhat surprising, as we applied conservative exclusion criteria (omitting patients with both primary and ancillary diagnoses of psychotic disorders, bipolar disorders, or manic episodes before the index episode) along with strict criteria for diagnostic change, considering only primary diagnoses, an approach not reported in previous studies. As noted, requiring at least two recorded diagnoses separated by ≥ 1 year in our sensitivity analysis reduced the cumulative incidence to 9.8%, which is more consistent with previous findings but still higher than in most other studies. Furthermore, since previous studies have not described the use of such a confirmation method, it can be assumed that their reported conversion rates might have been lower if this approach had been applied.

Few previous studies have examined a transition to psychotic disorders. A Finnish study by Baryshnikov et al. reported that 6.5% of patients converted to schizophrenia and 4.6% to schizoaffective disorder during a follow‐up period of up to 15 years [6]. These conversion rates are higher than those observed in our study, which is noteworthy considering that Baryshnikov et al. included only patients who were both diagnosed and experienced a diagnostic shift during inpatient care, thereby omitting potential diagnostic shifts occurring in outpatient settings.

In our sample, delusional disorder was more frequently diagnosed than schizophrenia and schizoaffective disorder, a finding not previously reported in large‐scale studies. If, for some patients, psychotic depression represents an initial manifestation of delusional disorder (in which functioning, apart from the delusion, is not markedly impaired), this challenges the long‐standing notion that the presence of psychotic symptoms automatically denotes a severe depressive episode. It also supports the ICD‐11 revision allowing psychotic depression to be classified as of moderate severity [1, 20]. Another possible interpretation is that preserved functioning in delusional disorder may delay recognition of delusional symptoms until depressive symptoms emerge, at which point the delusions are observed but misattributed to psychotic depression.

The most common subcategories of psychotic disorders were acute and transient psychotic disorders and other psychotic disorders. Both categories may represent a range of conditions, including a new episode of psychotic depression, the onset of a psychotic disorder such as schizophrenia, schizoaffective disorder, or delusional disorder, or transient psychotic symptoms not indicative of a persistent condition. The latter appears most common, as reflected by the marked decline in cumulative incidence observed in the sensitivity analysis: acute and transient psychotic disorders declined from 5.1% to 1.4%, and other psychotic disorders from 8.9% to 3.6%. Among the remaining cases, a significant proportion likely represents specific psychotic conditions, which may contribute to the underdiagnosis of these disorders.

We should also mention a recently published study by Nguyen et al., which, although primarily focused on the genetics of psychotic depression, also reported crude conversion rates based on Swedish and Danish registry data [21]. The reported rates were 17.4% for bipolar disorders, 15.9% for schizophrenia or schizoaffective disorder, and 21.9% for other psychotic disorders (defined as all other F2 diagnoses). However, interpretation is limited by the inclusion of individuals already diagnosed with these conditions prior to their psychotic depression diagnosis (3.1%, 3.6%, and 11.7%, respectively), unclear follow‐up durations for each national cohort, and the use of multiple diagnostic systems over time. The study found notable differences between countries: bipolar disorders (21.5% vs. 12.3%) and other psychotic disorders (26.8% vs. 15.9%) were more common in Sweden than in Denmark, whereas schizophrenia or schizoaffective disorder was more frequently diagnosed in Denmark (19.1% vs. 13.3%). These patterns align with our findings, in which bipolar disorders were more prevalent than previously reported, while schizophrenia and schizoaffective disorder were less frequent compared to Finnish and Danish cohorts [6, 21]. This may be partly explained by the fact that our Swedish cohort was drawn from the same registers used by Nguyen et al., although our follow‐up extended to 2021 (rather than 2013) and individuals with prior diagnoses of bipolar or psychotic disorders were excluded. While these findings may reflect true epidemiological differences, they could also result from variations in local diagnostic practices. Further research involving detailed reviews of medical records from large numbers of cases could lead to a better understanding of how these diagnoses are assigned and whether the observed differences represent genuine variation or reflect local diagnostic traditions.

This study did not aim to identify risk factors for diagnostic transition. However, our findings align with some of what is known regarding the development of schizophrenia and bipolar disorder. Schizophrenia has a peak onset at ages 21–25 in men, and two peaks in women: 25–30 and after age 45 [22]. Similarly, we found that transitions to all categories of psychotic disorders (including schizophrenia) were more common in males aged 0–25 and females aged 51–75. In bipolar disorder, inpatient care at the index episode is a well‐established predictor of transition from unipolar depression (including non‐psychotic depression), and we observed a higher cumulative incidence of transition to both bipolar and psychotic disorders among patients diagnosed during inpatient treatment [2, 3, 4]. Previous research has identified family history and female sex as predictors of transition from unipolar depression to bipolar disorder, and younger age and male sex as predictors of transition to schizophrenia [2, 3, 6]. Whether these apply specifically to psychotic depression is unclear and warrants further investigation, as such knowledge could facilitate accurate diagnosis and guide treatment decisions. Antipsychotics, a first‐line treatment for schizophrenia, have limited evidence supporting their use in psychotic depression, especially in the maintenance phase, and when used, they are typically prescribed as an adjunct to antidepressants [8, 9, 10, 23]. Given that antipsychotics are associated with several severe potential side effects, it is important to identify patients most likely to benefit from their use [11]. Antidepressants, on the other hand, are controversial in bipolar disorder due to concerns about both efficacy and safety, underscoring the clinical importance of recognizing that a substantial proportion of patients with psychotic depression will later receive a bipolar disorder diagnosis [24, 25].

Establishing the correct diagnosis is also important for prognostic assessment. Schizophrenia is associated with a poorer outcome than psychotic depression across several domains, including occupational functioning, severity of psychotic symptoms, and relapse rate [12]. Early intervention may improve long‐term outcomes in schizophrenia, underscoring the importance of recognizing that a substantial proportion of patients initially diagnosed with psychotic depression may later be rediagnosed with schizophrenia [13]. Whether prognosis differs between schizophrenia patients with versus without psychotic depression at onset remains unclear. In our study, the cumulative incidence of men aged 26–50 years at the index episode (e.g., after the typical peak onset age for schizophrenia) transitioning to schizophrenia was relatively high at 3.3%. As earlier onset has been linked to poorer outcomes, these patients may have a more favorable prognosis, although this requires further study [26].

In nearly all diagnostic categories, younger patients had a higher conversion rate than older patients. If older patients have a lower conversion rate (e.g., higher diagnostic stability of psychotic depression), this could influence treatment outcomes. In a previous study by our research group, we examined the association between maintenance electroconvulsive therapy (ECT) and the composite risk of hospital readmission and completed suicide in patients with psychotic depression, stratified by age (≤ 65 and > 65 years). Among older patients, maintenance ECT was statistically significantly associated with a lower risk, whereas no such association was observed in younger patients [27]. A higher diagnostic stability of psychotic depression in elderly patients may have contributed to these differing results.

A strength of this study is the inclusion of a large patient sample with a long follow‐up period, during which the same diagnostic system (ICD‐10) was consistently used. This was made possible by access to high‐quality Swedish registries with broad coverage. However, our study also has some limitations that should be considered. First, undiagnosed cases (e.g., patients not seeking care) are missing. Second, about half of the incident cases were diagnosed in outpatient care, but the Swedish Outpatient Register only covers about 80%, mainly due to missing data from private healthcare providers [14]. It remains unknown whether patients diagnosed in private settings differ systematically in terms of age, sex, or socioeconomic status; if such differences exist, they could introduce bias into the analyses. Moreover, we cannot access data at all from primary healthcare [14]. Based on our clinical experience, private psychiatric care in Sweden is predominantly used for nonpsychotic conditions. Consequently, we assume that well below 20% of patients diagnosed with psychotic depression in outpatient care are missing due to the lack of data from private healthcare. Regarding missing data from primary care, we find it highly unlikely that a substantial number of patients would be diagnosed with psychotic depression and subsequently managed exclusively within that setting. Third, although we implemented a “wash‐out” period of at least 4 years in outpatient care and 8 years in inpatient care to ensure no registered diagnosis of a preexisting psychotic or bipolar disorder before the index episode of psychotic depression, we cannot exclude the possibility that such episodes occurred earlier. Furthermore, it is possible that symptoms of prior hypomanic or manic episodes went unrecognized by the assessing clinician, potentially leading to misclassification. Fourth, the reliability of psychiatric diagnoses may represent an additional limitation. It is not uncommon for patients to experience symptoms for over a decade before receiving a diagnosis of bipolar disorder type 2 [28]. On the other hand, up to 40% of individuals with borderline personality disorder are misdiagnosed with bipolar disorder type 1 or type 2 [28]. In terms of psychotic disorders, the most frequently recorded categories in this study were the unspecified categories of acute and transient psychotic disorders and other psychotic disorders. These categories may encompass specific conditions such as schizophrenia or schizoaffective disorder, potentially leading to underdiagnosis of these more specific diagnoses. At the same time, there may be concerns regarding the reliability of specific psychotic disorder diagnoses as well. A Spanish registry‐based study by Lopez‐Castroman et al., including 26,163 patients initially diagnosed with schizophrenia, found that approximately 35% had their diagnosis revised within 48 months, most commonly within the first 6 months [29]. To address the risk of overestimating diagnostic transition in uncertain cases, we conducted a sensitivity analysis requiring the same diagnosis to be recorded at least twice, with ≥ 1 year between the first and last occurrence. A more stringent analysis excluded patients who, after their first transitional diagnosis, were later rediagnosed with psychotic depression, yielding only small differences for most diagnostic categories. However, this sensitivity analysis conditions not only on diagnostic stability but also on factors influencing the likelihood of returning for subsequent treatment; for example, patients who refuse further hospital care or who die after the initial diagnosis would not be detected. Overall, both underdiagnosis and misdiagnosis for psychotic and bipolar disorders remain possible.

In summary, our study reveals a high cumulative incidence of diagnostic shift after a first episode of psychotic unipolar depression. Approximately 20%–30% of patients in Sweden are expected to have their diagnosis changed to a bipolar or psychotic disorder within 17 years of their initial diagnosis. This finding has clinical implications, as prognosis and treatment guidelines differ between these disorders. Further research is needed to identify potential risk factors for diagnostic conversion, allowing for better assessment of at‐risk patients, which in turn could improve prognostic evaluations and treatment strategies. There is also a need to clarify whether the higher conversion rate to bipolar disorders in Sweden and to schizophrenia or schizoaffective disorder in Denmark and Finland reflects true epidemiological differences or varying local diagnostic traditions.

## Author Contributions

**Ahmed Al‐Wandi:** conceptualization, data curation, formal analysis, funding acquisition, investigation, methodology, project administration, software, validation, visualization, writing – original draft, writing – review and editing. **Mikael Landén:** conceptualization, methodology, supervision, validation, writing – review and editing. **Axel Nordenskjöld:** conceptualization, data curation, funding acquisition, investigation, methodology, project administration, resources, software, supervision, validation, writing – review and editing. All authors are accountable for all aspects of the work.

## Funding

This study was supported by the Örebro Regional Council.

## Ethics Statement

Approval for this study was granted by the regional ethical review board in Uppsala, Sweden (2014/174), and the Swedish Ethical Review Authority (2020‐05154 and 2021‐03815). Informed consent was waived because this was a registry‐based study in which the participants could not be identified at any time.

## Conflicts of Interest

The authors declare no conflicts of interest.

## Data Availability

The data that support the findings of this study are available on request from the corresponding author. The data are not publicly available due to privacy or ethical restrictions.

## Appendix A See Table A1

**TABLE A1:** Disorders and corresponding ICD‐10 codes.

Disorder	ICD‐10 codes
Psychotic unipolar depression	F32.3 and F33.3
Psychotic disorders	F04, F06.2, F07.9, F10.6, F20, F22‐F25, F28‐29
Bipolar disorder	F30–F31
Schizophrenia	F20^a^
Delusional disorder	F22
Schizoaffective disorder	F25
Acute and transient psychotic disorders	F23
Other psychotic disorders	F04, F06.2, F07.9, F10.6, F24, F28‐29
Bipolar disorder type 1	F30.1, F30.2, F30.8, F30.9, F31.1, F31.2

Abbreviation: ICD‐10 = International Classification of Diseases, 10th edition.

^a^ All ICD‐10 codes after the initial prefix are included, for example, F20 includes F20.0, F20.1, and so forth.

## Appendix B See Tables B1 and B2

**TABLE B1:** Conversion rate estimates (main analysis): 1 minus Kaplan–Meier, cumulative incidence function, and crude proportions.

Age	All	Male	Female
*Psychotic and bipolar disorders combined*			
All	1‐KM: 31.6%, CIF: 28.8%, Prop: 27.9%	1‐KM: 30.3%, CIF: 27.6%, Prop: 26.6%	1‐KM: 32.6%, CIF: 29.8%, Prop: 28.9%
0–25 years	1‐KM: 38.7%, CIF: 38.3%, Prop: 36.5%	1‐KM: 44.4%, CIF: 43.6%, Prop: 38.2%	1‐KM: 35.8%, CIF: 35.6%, Prop: 35.2%
26–50 years	1‐KM: 37.2%, CIF: 36.5%, Prop: 35.6%	1‐KM: 35.5%, CIF: 34.8%, Prop: 34.2%	1‐KM: 38.5%, CIF: 37.8%, Prop: 36.7%
51–75 years	1‐KM: 26.0%, CIF: 23.3%, Prop: 22.4%	1‐KM: 22.2%, CIF: 19.7%, Prop: 19.1%	1‐KM: 29.2%, CIF: 26.5%, Prop: 25.3%
> 75 years	1‐KM: 15.7%, CIF: 10.5%, Prop: 10.5%	1‐KM: 16.4%, CIF: 9.9%, Prop: 9.9%	1‐KM: 15.5%, CIF: 10.8%, Prop: 10.8%
*Psychotic disorders*			
All	1‐KM: 19.2%, CIF: 17.5%, Prop: 16.9%	1‐KM: 18.8%, CIF: 17.3%, Prop: 16.8%	1‐KM: 19.4%, CIF: 17.7%, Prop: 16.9%
0–25 years	1‐KM: 25.0%, CIF: 24.8%, Prop: 23.9%	1‐KM: 29.8%, CIF: 29.4%, Prop: 28.8%	1‐KM: 21.8%, CIF: 21.5%, Prop: 20.5%
26–50 years	1‐KM: 24.4%, CIF: 23.9%, Prop: 23.0%	1‐KM: 24.3%, CIF: 23.8%, Prop: 23.3%	1‐KM: 24.5%, CIF: 24.1%, Prop: 22.8%
51–75 years	1‐KM: 12.9%, CIF: 11.5%, Prop: 11.1%	1‐KM: 10.7%, CIF: 9.5%, Prop: 9.1%	1‐KM: 14.7%, CIF: 13.3%, Prop: 12.9%
> 75 years	1‐KM: 10.4%, CIF: 6.8%, Prop: 6.8%	1‐KM: 9.9%, CIF: 5.8%, Prop: 5.8%	1‐KM: 10.7%, CIF: 7.3%, Prop: 7.2%
*Bipolar disorder*			
All	1‐KM: 16.2%, CIF: 14.7%, Prop: 14.1%	1‐KM: 14.7%, CIF: 13.2%, Prop: 12.4%	1‐KM: 17.4%, CIF: 15.8%, Prop: 15.4%
0–25 years	1‐KM: 21.3%, CIF: 20.9%, Prop: 19.1%	1‐KM: 19.3%, CIF: 18.6%, Prop: 14.6%	1‐KM: 22.9%, CIF: 22.7%, Prop: 22.3%
26–50 years	1‐KM: 17.5%, CIF: 17.2%, Prop: 16.8%	1‐KM: 15.3%, CIF: 14.9%, Prop: 14.6%	1‐KM: 19.3%, CIF: 19.0%, Prop: 18.5%
51–75 years	1‐KM: 14.9%, CIF: 13.3%, Prop: 12.8%	1‐KM: 13.5%, CIF: 11.8%, Prop: 11.5%	1‐KM: 16.2%, CIF: 14.5%, Prop: 13.9%
> 75 years	1‐KM: 6.6%, CIF: 4.2%, Prop: 4.2%	1‐KM: 6.5%, CIF: 4.2%, Prop: 4.2%	1‐KM: 6.6%, CIF: 4.2%, Prop: 4.2%
*Schizophrenia*			
All	1‐KM: 3.7%, CIF: 3.3%, Prop: 3.2%	1‐KM: 4.5%, CIF: 4.1%, Prop: 4.0%	1‐KM: 3.1%, CIF: 2.7%, Prop: 2.6%
0–25 years	1‐KM: 7.6%, CIF: 7.5%, Prop: 7.0%	1‐KM: 11.3%, CIF: 11.0%, Prop: 10.6%	1‐KM: 5.1%, CIF: 5.0%, Prop: 4.4%
26–50 years	1‐KM: 5.4%, CIF: 5.2%, Prop: 5.0%	1‐KM: 6.5%, CIF: 6.3%, Prop: 6.2%	1‐KM: 4.5%, CIF: 4.4%, Prop: 4.1%
51–75 years	1‐KM: 1.1%, CIF: 0.9%, Prop: 0.9%	1‐KM: 0.7%, CIF: 0.6%, Prop: 0.5%	1‐KM: 1.4%, CIF: 1.2%, Prop: 1.2%
> 75 years	No cases	No cases	No cases
*Delusional disorder*			
All	1‐KM: 5.5%, CIF: 5.0%, Prop: 4.7%	1‐KM: 5.6%, CIF: 5.0%, Prop: 4.9%	1‐KM: 5.5%, CIF: 4.9%, Prop: 4.6%
0–25 years	1‐KM: 3.3%, CIF: 3.2%, Prop: 3.2%	1‐KM: 4.1%, CIF: 4.0%, Prop: 4.0%	1‐KM: 2.7%, CIF: 2.7%, Prop: 2.6%
26–50 years	1‐KM: 5.9%, CIF: 5.8%, Prop: 5.6%	1‐KM: 6.6%, CIF: 6.4%, Prop: 6.3%	1‐KM: 5.4%, CIF: 5.3%, Prop: 5.0%
51–75 years	1‐KM: 6.4%, CIF: 5.5%, Prop: 5.0%	1‐KM: 5.2%, CIF: 4.4%, Prop: 4.2%	1‐KM: 7.3%, CIF: 6.4%, Prop: 5.8%
> 75 years	1‐KM: 4.4%, CIF: 2.7%, Prop: 2.7%	1‐KM: 5.7%, CIF: 3.2%, Prop: 3.2%	1‐KM: 4.0%, CIF: 2.5%, Prop: 2.5%
*Schizoaffective disorder*			
All	1‐KM: 4.3%, CIF: 3.7%, Prop: 3.5%	1‐KM: 3.8%, CIF: 3.3%, Prop: 3.1%	1‐KM: 4.6%, CIF: 4.1%, Prop: 3.8%
0–25 years	1‐KM: 7.3%, CIF: 7.2%, Prop: 7.0%	1‐KM: 8.1%, CIF: 7.9%, Prop: 7.3%	1‐KM: 6.9%, CIF: 6.8%, Prop: 6.7%
26–50 years	1‐KM: 5.9%, CIF: 5.7%, Prop: 5.3%	1‐KM: 4.8%, CIF: 4.6%, Prop: 4.3%	1‐KM: 6.8%, CIF: 6.6%, Prop: 6.0%
51–75 years	1‐KM: 1.9%, CIF: 1.6%, Prop: 1.5%	1‐KM: 1.6%, CIF: 1.3%, Prop: 1.1%	1‐KM: 2.1%, CIF: 1.8%, Prop: 1.7%
> 75 years	1‐KM: 0.2%, CIF: 0.1%, Prop: 0.1%	No cases	1‐KM: 0.2%, CIF: 0.2%, Prop: 0.2%
*Acute and transient psychotic disorders*			
All	1‐KM: 5.8%, CIF: 5.1%, Prop: 4.7%	1‐KM: 4.8%, CIF: 4.3%, Prop: 3.9%	1‐KM: 6.5%, CIF: 5.7%, Prop: 5.3%
0–25 years	1‐KM: 10.4%, CIF: 10.2%, Prop: 8.9%	1‐KM: 11.0%, CIF: 10.8%, Prop: 9.0%	1‐KM: 10.0%, CIF: 9.8%, Prop: 8.9%
26–50 years	1‐KM: 7.3%, CIF: 7.1%, Prop: 6.6%	1‐KM: 6.0%, CIF: 5.8%, Prop: 5.4%	1‐KM: 8.2%, CIF: 8.0%, Prop: 7.5%
51–75 years	1‐KM: 2.7%, CIF: 2.3%, Prop: 2.1%	1‐KM: 1.6%, CIF: 1.4%, Prop: 1.4%	1‐KM: 3.7%, CIF: 3.1%, Prop: 2.8%
> 75 years	1‐KM: 2.2%, CIF: 1.7%, Prop: 1.7%	1‐KM: 1.4%, CIF: 1.3%, Prop: 1.3%	1‐KM: 2.5%, CIF: 1.8%, Prop: 1.8%
*Other psychotic disorders*			
All	1‐KM: 9.8%, CIF: 8.9%, Prop: 8.5%	1‐KM: 10.0%, CIF: 9.1%, Prop: 8.9%	1‐KM: 9.7%, CIF: 8.7%, Prop: 8.2%
0–25 years	1‐KM: 15.1%, CIF: 14.8%, Prop: 14.4%	1‐KM: 17.6%, CIF: 17.3%, Prop: 17.0%	1‐KM: 13.2%, CIF: 13.1%, Prop: 12.6%
26–50 years	1‐KM: 12.7%, CIF: 12.3%, Prop: 11.7%	1‐KM: 13.6%, CIF: 13.3%, Prop: 13.0%	1‐KM: 11.9%, CIF: 11.6%, Prop: 10.7%
51–75 years	1‐KM: 5.5%, CIF: 4.9%, Prop: 4.8%	1‐KM: 4.3%, CIF: 3.9%, Prop: 3.8%	1‐KM: 6.5%, CIF: 5.9%, Prop: 5.6%
> 75 years	1‐KM: 4.7%, CIF: 3.0%, Prop: 3.0%	1‐KM: 3.6%, CIF: 1.9%, Prop: 1.9%	1‐KM: 5.1%, CIF: 3.6%, Prop: 3.5%
*Bipolar disorder type 1*			
All	1‐KM: 3.3%, CIF: 2.9%, Prop: 2.8%	1‐KM: 3.6%, CIF: 3.1%, Prop: 2.9%	1‐KM: 3.1%, CIF: 2.8%, Prop: 2.7%
0–25 years	1‐KM: 5.1%, CIF: 5.0%, Prop: 4.3%	1‐KM: 6.5%, CIF: 6.2%, Prop: 4.5%	1‐KM: 4.4%, CIF: 4.3%, Prop: 4.1%
26–50 years	1‐KM: 3.6%, CIF: 3.5%, Prop: 3.4%	1‐KM: 3.5%, CIF: 3.4%, Prop: 3.4%	1‐KM: 3.7%, CIF: 3.6%, Prop: 3.4%
51–75 years	1‐KM: 2.5%, CIF: 2.2%, Prop: 2.2%	1‐KM: 2.7%, CIF: 2.4%, Prop: 2.4%	1‐KM: 2.4%, CIF: 2.1%, Prop: 2.1%
> 75 years	1‐KM: 1.2%, CIF: 0.9%, Prop: 0.9%	1‐KM: 1.9%, CIF: 1.3%, Prop: 1.3%	1‐KM: 0.9%, CIF: 0.8%, Prop: 0.8%

Abbreviations: 1**‐**KM, 1 minus Kaplan–Meier estimator; CIF, cumulative incidence function; Prop, crude proportion.

**TABLE B2:** Conversion rate estimates (sensitivity analysis): 1 minus Kaplan–Meier, cumulative incidence function, and crude proportions, requiring ≥ 2 diagnoses at least 1 year apart.

Age	All	Male	Female
*Psychotic and bipolar disorders combined*			
All	1‐KM: 20.8%, CIF: 19.0%, Prop: 18.4%	1‐KM: 20.1%, CIF: 18.5%, Prop: 18.1%	1‐KM: 21.3%, CIF: 19.5%, Prop: 18.6%
0–25 years	1‐KM: 26.6%, CIF: 26.3%, Prop: 25.8%	1‐KM: 28.1%, CIF: 27.6%, Prop: 27.4%	1‐KM: 25.5%, CIF: 25.3%, Prop: 24.8%
26–50 years	1‐KM: 26.8%, CIF: 26.2%, Prop: 25.3%	1‐KM: 26.5%, CIF: 25.9%, Prop: 25.5%	1‐KM: 26.9%, CIF: 26.4%, Prop: 25.1%
51–75 years	1‐KM: 15.6%, CIF: 14.0%, Prop: 13.4%	1‐KM: 12.8%, CIF: 11.5%, Prop: 11.1%	1‐KM: 18.1%, CIF: 16.3%, Prop: 15.4%
> 75 years	1‐KM: 4.5%, CIF: 3.3%, Prop: 3.3%	1‐KM: 5.5%, CIF: 3.8%, Prop: 3.8%	1‐KM: 4.0%, CIF: 3.1%, Prop: 3.1%
*Psychotic disorders*			
All	1‐KM: 10.9%, CIF: 10.0%, Prop: 9.6%	1‐KM: 11.2%, CIF: 10.3%, Prop: 10.0%	1‐KM: 10.8%, CIF: 9.8%, Prop: 9.3%
0–25 years	1‐KM: 15.5%, CIF: 15.3%, Prop: 15.0%	1‐KM: 19.3%, CIF: 19.1%, Prop: 18.9%	1‐KM: 12.8%, CIF: 12.7%, Prop: 12.3%
26–50 years	1‐KM: 15.4%, CIF: 15.0%, Prop: 14.4%	1‐KM: 16.3%, CIF: 15.9%, Prop: 15.4%	1‐KM: 14.7%, CIF: 14.4%, Prop: 13.6%
51–75 years	1‐KM: 6.3%, CIF: 5.6%, Prop: 5.3%	1‐KM: 4.4%, CIF: 4.0%, Prop: 3.8%	1‐KM: 7.9%, CIF: 7.1%, Prop: 6.6%
> 75 years	1‐KM: 1.7%, CIF: 1.5%, Prop: 1.5%	1‐KM: 1.6%, CIF: 1.3%, Prop: 1.3%	1‐KM: 1.8%, CIF: 1.5%, Prop: 1.5%
*Bipolar disorder*			
All	1‐KM: 10.7%, CIF: 9.8%, Prop: 9.4%	1‐KM: 9.5%, CIF: 8.6%, Prop: 8.4%	1‐KM: 11.7%, CIF: 10.6%, Prop: 10.1%
0–25 years	1‐KM: 13.0%, CIF: 12.7%, Prop: 12.5%	1‐KM: 10.2%, CIF: 10.0%, Prop: 9.9%	1‐KM: 14.8%, CIF: 14.6%, Prop: 14.3%
26–50 years	1‐KM: 12.8%, CIF: 12.5%, Prop: 12.1%	1‐KM: 11.1%, CIF: 10.8%, Prop: 10.7%	1‐KM: 14.0%, CIF: 13.8%, Prop: 13.1%
51–75 years	1‐KM: 9.4%, CIF: 8.4%, Prop: 8.0%	1‐KM: 8.4%, CIF: 7.4%, Prop: 7.1%	1‐KM: 10.3%, CIF: 9.3%, Prop: 8.8%
> 75 years	1‐KM: 3.6%, CIF: 1.9%, Prop: 1.9%	1‐KM: 3.9%, CIF: 2.6%, Prop: 2.6%	1‐KM: 3.2%, CIF: 1.6%, Prop: 1.5%
*Schizophrenia*			
All	1‐KM: 2.3%, CIF: 2.0%, Prop: 1.9%	1‐KM: 2.8%, CIF: 2.5%, Prop: 2.4%	1‐KM: 1.9%, CIF: 1.7%, Prop: 1.5%
0–25 years	1‐KM: 4.3%, CIF: 4.3%, Prop: 4.3%	1‐KM: 6.7%, CIF: 6.6%, Prop: 6.6%	1‐KM: 2.7%, CIF: 2.6%, Prop: 2.6%
26–50 years	1‐KM: 3.4%, CIF: 3.3%, Prop: 3.0%	1‐KM: 4.1%, CIF: 4.0%, Prop: 3.8%	1‐KM: 2.8%, CIF: 2.7%, Prop: 2.4%
51–75 years	1‐KM: 0.7%, CIF: 0.6%, Prop: 0.6%	1‐KM: 0.4%, CIF: 0.3%, Prop: 0.3%	1‐KM: 1.0%, CIF: 0.9%, Prop: 0.8%
> 75 years	No cases	No cases	No cases
*Delusional disorder*			
All	1‐KM: 2.6%, CIF: 2.4%, Prop: 2.3%	1‐KM: 2.9%, CIF: 2.7%, Prop: 2.6%	1‐KM: 2.4%, CIF: 2.2%, Prop: 2.1%
0–25 years	1‐KM: 0.7%, CIF: 0.7%, Prop: 0.7%	1‐KM: 1.2%, CIF: 1.2%, Prop: 1.2%	1‐KM: 0.3%, CIF: 0.3%, Prop: 0.3%
26–50 years	1‐KM: 3.2%, CIF: 3.1%, Prop: 3.0%	1‐KM: 3.9%, CIF: 3.8%, Prop: 3.7%	1‐KM: 2.6%, CIF: 2.5%, Prop: 2.4%
51–75 years	1‐KM: 3.2%, CIF: 2.8%, Prop: 2.7%	1‐KM: 2.7%, CIF: 2.4%, Prop: 2.2%	1‐KM: 3.6%, CIF: 3.2%, Prop: 3.1%
> 75 years	1‐KM: 1.0%, CIF: 0.8%, Prop: 0.8%	1‐KM: 1.2%, CIF: 1.0%, Prop: 1.0%	1‐KM: 0.9%, CIF: 0.8%, Prop: 0.8%
*Schizoaffective disorder*			
All	1‐KM: 3.1%, CIF: 2.7%, Prop: 2.5%	1‐KM: 2.9%, CIF: 2.5%, Prop: 2.2%	1‐KM: 3.2%, CIF: 2.8%, Prop: 2.7%
0–25 years	1‐KM: 5.1%, CIF: 5.0%, Prop: 4.8%	1‐KM: 5.2%, CIF: 5.1%, Prop: 5.0%	1‐KM: 5.0%, CIF: 4.9%, Prop: 4.8%
26–50 years	1‐KM: 4.2%, CIF: 4.1%, Prop: 3.7%	1‐KM: 3.7%, CIF: 3.5%, Prop: 3.0%	1‐KM: 4.7%, CIF: 4.6%, Prop: 4.3%
51–75 years	1‐KM: 1.4%, CIF: 1.2%, Prop: 1.0%	1‐KM: 1.4%, CIF: 1.2%, Prop: 0.9%	1‐KM: 1.4%, CIF: 1.2%, Prop: 1.1%
> 75 years	1‐KM: 0.2%, CIF: 0.1%, Prop: 0.1%	No cases	1‐KM: 0.2%, CIF: 0.2%, Prop: 0.2%
*Acute and transient psychotic disorders*			
All	1‐KM: 1.5%, CIF: 1.4%, Prop: 1.3%	1‐KM: 1.0%, CIF: 0.9%, Prop: 0.9%	1‐KM: 2.0%, CIF: 1.8%, Prop: 1.7%
0–25 years	1‐KM: 3.0%, CIF: 3.0%, Prop: 2.7%	1‐KM: 2.4%, CIF: 2.4%, Prop: 2.4%	1‐KM: 3.4%, CIF: 3.3%, Prop: 3.0%
26–50 years	1‐KM: 2.3%, CIF: 2.3%, Prop: 2.1%	1‐KM: 1.3%, CIF: 1.3%, Prop: 1.3%	1‐KM: 3.1%, CIF: 3.0%, Prop: 2.8%
51–75 years	1‐KM: 0.4%, CIF: 0.4%, Prop: 0.4%	1‐KM: 0.2%, CIF: 0.2%, Prop: 0.2%	1‐KM: 0.5%, CIF: 0.5%, Prop: 0.5%
> 75 years	1‐KM: 0.1%, CIF: 0.1%, Prop: 0.1%	No cases	1‐KM: 0.2%, CIF: 0.2%, Prop: 0.2%
*Other psychotic disorders*			
All	1‐KM: 3.9%, CIF: 3.6%, Prop: 3.4%	1‐KM: 4.1%, CIF: 3.8%, Prop: 3.7%	1‐KM: 3.7%, CIF: 3.4%, Prop: 3.2%
0–25 years	1‐KM: 7.0%, CIF: 6.9%, Prop: 6.7%	1‐KM: 8.6%, CIF: 8.5%, Prop: 8.5%	1‐KM: 5.8%, CIF: 5.8%, Prop: 5.4%
26–50 years	1‐KM: 5.7%, CIF: 5.6%, Prop: 5.3%	1‐KM: 6.5%, CIF: 6.3%, Prop: 6.1%	1‐KM: 5.1%, CIF: 5.0%, Prop: 4.7%
51–75 years	1‐KM: 1.3%, CIF: 1.2%, Prop: 1.2%	1‐KM: 0.6%, CIF: 0.5%, Prop: 0.5%	1‐KM: 2.0%, CIF: 1.9%, Prop: 1.9%
> 75 years	1‐KM: 0.4%, CIF: 0.3%, Prop: 0.3%	1‐KM: 0.4%, CIF: 0.3%, Prop: 0.3%	1‐KM: 0.3%, CIF: 0.3%, Prop: 0.3%

Abbreviations: 1‐KM, 1 minus Kaplan–Meier estimator; CIF, cumulative incidence function; Prop, crude proportion.

## Appendix C See Table C1

**TABLE C1:** Sensitivity analysis.

Age	All	Male	Female	All	Male	Female
*Psychotic and bipolar disorders*				Psychotic disorders		
All	19.0% (18.1–20.0)^a^	18.5% (17.2–19.8)	19.5% (18.2–20.7)	10.0% (9.3–10.7)	10.3% (9.3–11.4)	9.8% (8.9–10.8)
0–25 years	26.3% (23.6–29.1)	27.6% (23.4–32.0)	25.3% (21.8–28.9)	15.3% (13.1–17.6)	19.1% (15.4–23.0)	12.7% (10.1–15.6)
26–50 years	26.2% (24.6–27.9)	25.9% (23.5–28.3)	26.4% (24.2–28.7)	15.0% (13.7–16.4)	15.9% (13.9–18.0)	14.4% (12.6–16.2)
51–75 years	14.0% (12.7–15.4)	11.5% (9.8–13.3)	16.3% (14.3–18.4)	5.6% (4.7–6.6)	4.0% (3.0–5.1)	7.1% (5.7–8.6)
> 75 years	3.3% (2.3–4.6)	3.8% (2.1–6.4)	3.1% (1.9–4.6)	1.5% (0.8–2.4)	1.3% (0.4–3.1)	1.5% (0.8–2.7)
*Bipolar disorders*				*Schizophrenia*		
All	9.8% (9.1–10.5)	8.6% (7.7–9.7)	10.6% (9.7–11.6)	2.0% (1.7–2.4)	2.5% (2.0–3.1)	1.7% (1.3–2.1)
0–25 years	12.7% (10.8–14.9)	10.0% (7.3–13.1)	14.6% (11.9–17.6)	4.3% (3.1–5.6)	6.6% (4.5–9.2)	2.6% (1.6–4.1)
26–50 years	12.5% (11.3–13.7)	10.8% (9.2–12.6)	13.8% (12.1–15.5)	3.3% (2.6–4.0)	4.0% (3.0–5.2)	2.7% (1.9–3.8)
51–75 years	8.4% (7.4–9.5)	7.4% (6.0–9.0)	9.3% (7.8–10.9)	0.6% (0.4–1.0)	0.3% (0.1–0.8)	0.9% (0.5–1.5)
> 75 years	1.9% (1.2–3.0)	2.6% (1.2–4.8)	1.6% (0.8–2.9)	No cases	No cases	No cases
*Delusional disorder*				*Schizoaffective disorder*		
All	2.4% (2.1–2.8)	2.7% (2.1–3.3)	2.2% (1.8–2.7)	2.7% (2.3–3.1)	2.5% (1.9–3.2)	2.8% (2.4–3.4)
0–25 years	0.7% (0.3–1.3)	1.2% (0.4–2.6)	0.3% (0.1–1.1)	5.0% (3.8–6.5)	5.1% (3.3–7.6)	4.9% (3.4–6.9)
26–50 years	3.1% (2.5–3.8)	3.8% (2.9–4.9)	2.5% (1.8–3.4)	4.1% (3.4–4.9)	3.5% (2.5–4.9)	4.6% (3.6–5.7)
51–75 years	2.8% (2.2–3.5)	2.4% (1.6–3.4)	3.2% (2.4–4.3)	1.2% (0.8–1.8)	1.2% (0.5–2.2)	1.2% (0.7–2.0)
> 75 years	0.8% (0.4–1.6)	1.0% (0.3–2.6)	0.8% (0.3–1.7)	0.1% (0.0–0.6)	No cases	0.2% (0.0–0.8)
*Acute and transient psychotic disorders*				*Other psychotic disorders*		
All	1.4% (1.1–1.7)	0.9% (0.6–1.3)	1.8% (1.4–2.2)	3.6% (3.1–4.0)	3.8% (3.2–4.5)	3.4% (2.8–4.0)
0–25 years	3.0% (2.0–4.2)	2.4% (1.2–4.2)	3.3% (2.0–5.2)	6.9% (5.4–8.7)	8.5% (6.1–11.4)	5.8% (4.0–8.0)
26–50 years	2.3% (1.7–2.9)	1.3% (0.8–2.0)	3.0% (2.2–4.0)	5.6% (4.8–6.5)	6.3% (5.0–7.7)	5.0% (4.0–6.2)
51–75 years	0.4% (0.2–0.6)	0.2% (0.1–0.6)	0.5% (0.2–0.9)	1.2% (0.9–1.7)	0.5% (0.2–1.1)	1.9% (1.3–2.7)
> 75 years	0.1% (0.0–0.6)	No cases	0.2% (0.0–0.8)	0.3% (0.1–0.9)	0.3% (0.0–1.7)	0.3% (0.1–1.1)

*Note*: Cumulative incidence of diagnostic conversion, requiring at least two recorded diagnoses separated by ≥ 1 year, stratified by age and sex.

^a^ Values in parentheses indicate 95% confidence intervals.
